# Local Treatment of Chancres by Sulphate of Copper and Cyanuret of Mercury

**Published:** 1843-05

**Authors:** 


*Local Treatment of Chancres by Sulphate of Copper and Cyanuret of Mercury.* By Dr. Strohl.—The more rapidly primary syphilis is cured, the less likely are secondary symptoms to appear. The first object is most easily attained by cauterizing. Sometimes, however, this method is inapplicable; for instance, when the sore is very extensive, or much inflamed. In such cases, the author employs the sulphate of copper. The sores are dressed five or six times a day with charpie, which has been soaked in a solution of about a grain and a half of sulphate of copper to an ounce of water. Simple chancres, when thus treated, usually heal within twelve days. Dr. Strohl assures us, that he cures complicated chancres in an equally short time with an ointment composed of two grains of cyanuret of mercury to an ounce of axunge. This ointment is spread upon a piece of linen corresponding to the size of the sore. This dressing is apt to be painful at first; and it must occasionally be taken off, after it has been on for an hour or two, and the remedy must be applied in a weaker form. The pain is said to be most violent in half an hour or an hour, and frequently ceases entirely in two or three hours. When the chancre is extensive and painful, after the ointment has been on from four to ten hours, according to the sensibility of the patient, it is dressed with mercurial ointment, or opium cerate.

If the edges of the chancre have flattened, if the centre is cleaner, the pain less, and the suppuration healthy (which may occur after the first application of the cyanuret of mercury, but, at any rate, not later than the fourth), the treatment with the sulphate of copper is finished.—*Lond. Med. Gaz.*, Nov. 1842, from *Oesterr. Med. Wochenschrift.*

